# Cannabis Vaping Among Youth and Young Adults: a Scoping Review

**DOI:** 10.1007/s40429-022-00413-y

**Published:** 2022-05-07

**Authors:** Melissa B. Harrell, Stephanie L. Clendennen, Aslesha Sumbe, Kathleen R. Case, Dale S. Mantey, Sunaina Swan

**Affiliations:** 1 School of Public Health, University of Texas Health Science Center at Houston (UTHealth), 1616 Guadalupe St., Suite 6.300, Austin, TX 78701 USA; 2 Center for Research to Advance Community Health, UTHealth San Antonio, San Antonio, TX USA

**Keywords:** Cannabis, Vaping, Health effects, Epidemiology, Etiology, Regulation

## Abstract

**Purpose of Review:**

The purpose of this review was to describe the state-of-the-literature on research specific to cannabis vaping among youth and young adults.

**Recent Findings:**

Out of 1801 records identified, a total of 202 articles met eligibility criteria for inclusion in this review. Most of this literature (46.0% of studies) was specific to the health effects of cannabis vaping, particularly EVALI (e-cigarette and vaping associated lung injury). Other research areas identified in the review included the etiology (24.3%) and epidemiology (24.8%) of cannabis vaping, in addition to articles on regulation (8.4%) and marketing (5.5%) of the same.

**Summary:**

Cannabis vaping is increasingly common among youth and young adults and more prevalent is settings where recreational use for adults has been legalized. The literature documents a number of negative health effects of cannabis vaping for young people, along with risk factors and reasons for the same.

**Supplementary Information:**

The online version contains supplementary material available at 10.1007/s40429-022-00413-y.

## Introduction

The purpose of this review was to describe the state-of-the-literature regarding research specific to cannabis vaping among youth and young adults [1]. A recent meta-analysis found that among adolescents in the USA and Canada, lifetime, past-12 month, and 30-day prevalence of cannabis vaping increased by two- to seven-fold, from 2013 to 2020 [2•]. Preference for cannabis products, therefore, may be shifting from dried herb to cannabis oil (e.g., liquid tetrahydrocannabinol (THC), the primary psychoactive component in cannabis) — and, given the emergence [3] and predominance of e-cigarette use among young people now [4, 5], from smoking to vaping cannabis. Here, we provide a summary of results from 202 original investigations on this topic (see Supplementary Table 2), published between 2007 and 2021. These studies include information relevant to the epidemiology, health effects, etiology, marketing, and regulation of cannabis vaping and are applicable to young people less than 30 years old.

## Methods

This review was conducted using methods consistent with the Preferred Reporting Items for Systematic Review and Meta-Analysis for scoping reviews (PRISMA-ScR) [6]. A scoping review was deemed appropriate, as it focuses on an assessment of the potential size and scope of available literature. We categorize the available literature into relevant sub-topics and summarize the findings in the “Results” section.

### Search Strategy

Our search strategy was informed by recent published reviews of vaping among youth and young adults specific to the impact that nicotine vaping has on subsequent cannabis [7] or combustible tobacco product use [8–11]. Consistent with these reviews, the following databases were searched for studies: PubMed, Embase, and Web of Science. In addition, searches were conducted on the aforementioned reviews [7–11] and relevant summary reports [3, 12, 13] for additional studies, too. Keywords included: e-cigarettes, vaping, ENDS, JUUL; cannabis, marijuana, THC, CBD; adolescents, young adults. Keywords used for each database are provided in Supplementary Table 1. When available, we used subject headings (PubMed: MeSH; Embase: Emtree). Searches were conducted from January 2007 to December 2021. The initial date was chosen as it coincides with the appearance of the “modern” e-cigarette or vaping device on the international market [14].

### Study Selection

All studies retrieved through these searches were imported into software designed to support systematic reviews (https://www.covidence.org/) [15] and study selection was done there. There were five inclusion criteria. First, we included studies that examined cannabis vaping as a central topic or had findings about cannabis vaping. Second, study participants were youth (12–18 years old) and/or young adults (18–29 years old), or the study findings were relevant for young people (e.g., the unit of analysis was advertisements, but study findings were relevant for youth and/or young adults). Third, studies were original research; fourth, studies were full-text articles; and fifth, the articles were written in English. The reasons for exclusion included studies in which cannabis was not vaped, but only consumed in some other form; studies that only addressed vaping with substances other than cannabis (e.g., nicotine, flavors only); studies whose participants were not youth or young adults; animal studies; and, finally, articles that were not original research (e.g., editorials, commentaries, and review articles).

**Fig. 1:**
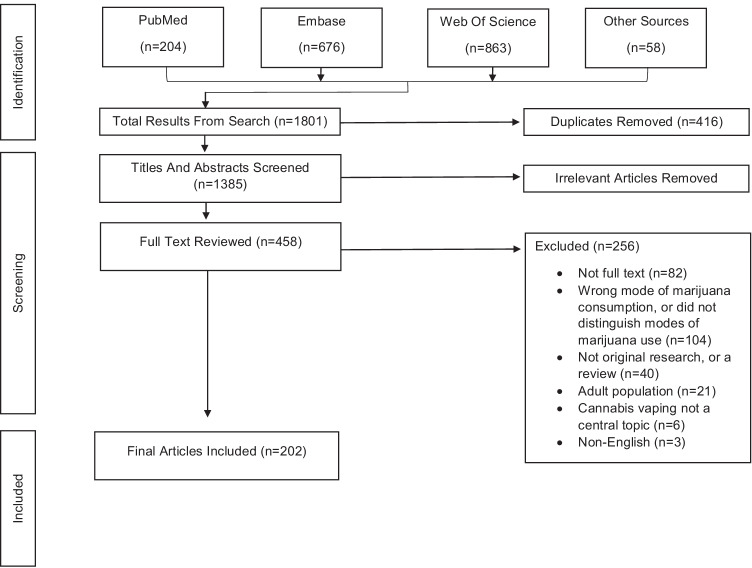
PRISMA flow diagram

A total of 1801 articles were initially identified. After removing 416 duplicates, 1385 titles and abstracts were independently reviewed by at least 2 of the 6 co-authors to determine their suitability for full-text screening. After removing 927 articles at this stage, each of 458 articles selected for full-text screening was again independently reviewed by at least 2 co-authors for inclusion. Any discrepancies between reviewers were discussed among all co-authors until consensus was reached. Ultimately, 202 articles were deemed relevant. Figure 1 is the PRISMA flow diagram for this process. Supplementary Table 2 provides a list of these 202 articles.

### Extraction

Data extracted from each study included the following: study design; study sub-topic, including epidemiology, health effects, etiology, marketing, and regulation; key findings about cannabis vaping within each sub-topic area; and, where relevant, study characteristics (e.g., location, participants’ age). Typical of a scoping review, no formal assessment of the quality of the eligible manuscripts was conducted, so information on the risk of bias that may be inherent in each of these studies is not presented here.

## Results

### Overview

No studies that met the inclusion criteria were published from 2007 to 2013. From 2013 onwards, the rate of publication increased exponentially, reaching a peak in 2020 (Supplementary Fig. 1) that was driven, in large part, by many articles (*n* = 70) on EVALI (e.g., case reports). Table 1 provides a summary of the number of articles reviewed here, by study design and sub-topic. Most studies were specific to health effects and etiology.

**Table 1:** Frequency of studies by study design and research area (*n* = 202)

Characteristic	*n*	(%)
Study designs^1^		
Case-reports	44	(21.8%)
Case-series	28	(13.9%)
Randomized controlled trials	4	(2.0%)
Observational	105	(52.0%)
Cross-sectional	*84*	*(41.6%)*
Cohort/longitudinal	*21*	*(10.4%)*
Qualitative	18	(8.9%)
Mixed methods^2^	2	(1.0%)
Meta-analysis	1	(0.5%)
Research areas^3^		
Health effects	93	(46.0%)
Etiology	49	(24.3%)
Epidemiology	50	(24.8%)
Regulatory policy	17	(8.4%)
Marketing/advertising	11	(5.5%)

^1^Categories are mutually exclusive. ^2^Mixed methods studies were cross-sectional + case series and longitudinal + qualitative. ^3^Categories are mutually inclusive

### Epidemiology

#### National Estimates

Annual prevalence estimates of cannabis vaping were available from Monitoring the Future (MTF) from 2017 to 2021 for youth (8th, 10th, and 12th grade) and from 2017 to 2021 for young adults (college students) and are shown in Supplementary Fig. 2 (lifetime use) and Supplementary Fig. 3 (past 30-day use) [4]. Lifetime use increased rapidly for both age groups from 2017 to 2020 then, for youth, declined in 2021. Among youth, lifetime prevalence rose from 8.5% in 2017 to 20.1% in 2020, then decreased to 15.9% in 2021. Among young adults, lifetime prevalence increased from 14.4% in 2017 to 34.6% in 2020. Notably, the doubling of past 30-day cannabis vaping observed among young adults (college students) from 2017 to 2018 was among the largest 1-year increase in any substance use ever recorded in the history of MTF (> 40 years) [4]. From 2018 to 2019, a similar doubling in the prevalence of past 30-day cannabis vaping was also observed for youth (8th, 10th, and 12th grade) [16]. Frequent vaping (defined as using ≥ 10 times in the last month) increased significantly among high school seniors from 2018 to 2019, too [17]. MTF data for young adults for 2021 were not available at this writing. (Note that the MTF publications that these prevalence estimates are derived from report data on “vaping marijuana” questions measured in the vaping section of the survey [4, 16]. One recent study shows that there is some discordance in prevalence estimates, if measures from the cannabis section on “using cannabis in a vaporizer” are used, instead [18]. Thus, researchers must take care when considering how best to ask questions about cannabis vaping and where to place them on a survey instrument [19].

Despite these alarming statistics, it is worth noting that, for each year (2017 to 2020), behavioral measure, and age group, the prevalence of cannabis vaping was less than the prevalence of nicotine vaping (data not shown) [4, 20–23]. For young adults, the prevalence of cannabis vaping was 20–30% less than that for nicotine vaping, while it was 50–60% less among youth. It is also worth reporting that from 2015 to 2018 (latest published data, among 12th graders only), cannabis smoking decreased, while cannabis vaping and the use of edibles increased [24]. From 2015 to 2018, for all students and among past-year cannabis users specifically, the prevalence of smoking cannabis was 2–3 times higher than the prevalence of vaping or use of edibles [24, 25].

This comparison across substances (nicotine vs. cannabis) and modes of consumption (smoking vs. vaping vs. edibles) helps to contextualize cannabis vaping among young people, demonstrating that, for now, it may not be as problematic as other substance use behaviors. However, given observed increases in recent years, efforts should continue to monitor cannabis vaping among young people in the future, and assessing modes of consumption may be a helpful way to guide prevention efforts [26]. Data on cannabis vaping are currently collected not only by MTF but also by the National Youth Tobacco Survey (NYTS) (youth only) [27–29], the Behavioral Risk Factor Surveillance System (BRFSS) (young adults only) [30, 31], and the Population Assessment of Tobacco Use and Health (PATH) study (youth and young adults) [32]. Each surveillance system offers unique insights into this phenomenon, given data it collects not only on cannabis vaping but also on other substance use behaviors and modes of cannabis consumption, too.

An analysis of PATH data from 2015 to 2016 for young adults (18–24 years old), for example, showed that lifetime or ever use of cigar products (e.g., cigarillos) to consume cannabis (i.e., blunt use) was 3 times more common than consuming cannabis by vaping or using a hookah to do so [32]. BRFSS surveys also include items on different modes of cannabis consumption (i.e., smoking, vaping, eating, and dabbing) [30] and can be used to compare nicotine vaping and cannabis vaping [31]. Analyses of BRFSS data from 2018 showed past 30-day dual nicotine and cannabis vaping was as common among young adults as past 30-day cannabis vaping alone [31]. Analyses of NYTS data from 2017 and 2018 showed that cannabis vaping was especially common for students who frequently use (i.e., use ≥ 20 days per month) e-cigarettes [27]. Surveillance data on lifetime or ever cannabis vaping on the 2016 [28], 2017 [29], and 2018 [29] NYTS surveys are consistent with the sustained increase reported in MTF, too. Two published studies [33, 34] specifically examined the possible impact of the COVID-19 epidemic on cannabis vaping among youth and young adults. One study showed that individual use increased, decreased, or stayed the same, depending on relevant risk factors (e.g., access, dependence) [33]. Another national study of more than 4000 adolescents and young adults showed only 6.8% of participants increased cannabis vaping during the pandemic, while 37% reduced or quit cannabis vaping, and 42.3% reported no change [34]. Though not explicitly stated by MTF, COVID-19 could be a reason for the leveling-off of past 30-day cannabis vaping from 2019 to 2021 among both age groups seen in MTF (Fig. 4).

#### Estimates by State

The prevalence of cannabis vaping among young people does vary by state, consistent with differences in local policies regarding age restrictions on use [35–43]. More information is provided below (see “Regulation” section). Data from the 2017 Healthy Kids Colorado survey, one of the first states to legalize recreational cannabis use for adults, showed that vaping was far less common as a mode of consumption among youth than smoking or ingesting cannabis (i.e., edible use) [44]. NYTS data from Florida (2015) [45] and North Carolina (2017) [46], where recreational cannabis use was and still is illegal, even for adults, showed 1 in 10 high students reported cannabis vaping.

#### International Estimates

Several studies identified through this review provided estimates of cannabis vaping among young people from other countries [47–53]. Data for 16 to 19 year-olds from the International Tobacco Control Policy Evaluation (ITC) study showed cannabis vaping is more prevalent in the USA, followed by Canada and England, consistent with regulatory policy in each country [51–53]. From 2017 to 2019, no significant differences in the prevalence of past 30-day use of a vaporizer to heat dried herb were observed within countries (England: 8.1% (2017), 12.5% (2018), 11.1% (2019); Canada: 15.9% (2017), 19.3% (2018), 19.1% (2019); USA: 20.6% (2017), 21.5% (2018), 23.0% (2019)). However, from 2017 to 2019, significant increases were observed for each country in the prevalence of past 30-day use of an e-cigarette to vape oil or liquid (England: 9.0% (2017), 14.6% (2018), 19.0% (2019); Canada: 12.9% (2017), 18.8% (2018), 25.9% (2019); USA: 24.2% (2017), 31.0% (2018), 52.1% (2019)). In 2017, for all three countries, vaping herb or vaping oil/liquid was the least common modes of cannabis consumption; yet, from 2017 to 2019, across all three countries, vaping oil/liquid increased more than other modes of consumption (vaping herb, smoking, and edible use) [51]. In addition, while nicotine vaping was also more prevalent than cannabis vaping across these countries [53], dual use was common [52]; and, in 2018, past 30-day cannabis users in the USA were more likely to report vaping cannabis than in Canada or England [53]. Findings from the COMPASS study in Canada before legalization in 2018 showed that ingesting and/or vaping cannabis were more often in addition to smoking cannabis, rather than as a replacement [49]. Follow-up studies from COMPASS that included data from 2017 to 2018 and 2018–2019 showed multiple modes of cannabis consumption (smoking, vaping, and/or ingesting) and increased frequency of the same were more common among high school students after legalization in 2018 [54–56]. Other studies from Finland [48] and Germany [50] show cannabis vaping among young people, at < 10%, may be less prevalent in these European countries than in England [51] though differences between studies by age and year of data collection make comparison difficult. Pooled estimates across 2016–2018 from one study of youth (14–15 years old) in New Zealand show cannabis vaping is less prevalent there, too [57]. Less than 10% of students reported using cannabis in the past 30-day days and, of these, only 7% reported exclusive cannabis vaping; 90% reported smoking cannabis, in contrast [57]. Estimates from other countries were not located in this review.

#### Use Trajectories

Several studies reported on developmental trajectories of use that describe the onset and progression of cannabis vaping across adolescence into young adulthood [58–63]. Several of these studies focused on cannabis vaping only [60–63], while others show cannabis vaping co-occurs with other substance use behaviors [58, 59]. Studies from California [60] and Texas [63] both show variability by sub-groups of young people: some young people start cannabis vaping in adolescence, while others begin in young adulthood; and some trajectories escalate quickly, while others escalate less rapidly. However, declines in use from adolescence into young adulthood are generally not observed [60, 63]. Taken together, these studies suggest a strong, positive relationship between age and cannabis vaping that may (or may not) peak in young adulthood. No studies reported on quit intentions or behaviors, providing scope for future research.

#### Special Populations

Several studies reported on use in special populations [64–69], like hospitalized adolescents [69], pregnant young adults [68], young adults with asthma [67], nightclub patrons [66], young people with inflammatory bowel disease [65], and young people in substance use treatment centers [64]. The reader is directed to these publications for further information. Data for all of these studies were collected between 2015 and 2020.

### Health Effects

#### EVALI

In Spring 2019, reports of e-cigarette, or vaping, associated lung injury (EVALI) began to emerge across the USA, with cases peaking in Summer 2019 [1, 2•]. Cases were characterized by respiratory, gastrointestinal, and/or constitutional symptoms, including cough, difficulty breathing, nausea, diarrhea, fever, chills, and/or weight loss [1, 3]. To date, the Centers for Disease Control and Prevention (CDC) has recorded 2807 EVALI cases and 68 deaths across 50 states, Puerto Rico and the US Virgin Islands. The severity of EVALI varies, with the most severe cases leading to extended ICU stays, lung transplants, and death [1, 2•]. Given the emergence of EVALI and the subsequent research that implicated vitamin E acetate found in some cannabis vape products in the outbreak, the majority of studies investigating the health effects of cannabis vaping (*n* = 93) focused on EVALI (*n* = 70), including 34 case reports [70–102], 25 case series [103–128], 8 cross-sectional studies [129•, 130–136], 1 longitudinal study [137], and 1 qualitative study [138]. One study included a case series of EVALI patients and a cross-sectional analysis of vaping devices; therefore, this was counted as both a case series and cross-sectional study [26].

The case series and case reports detailed symptoms at diagnosis including respiratory, gastrointestinal, and constitutional symptoms; and information on the treatment and outcomes of numerous patients with EVALI. Among the case reports (*n* = 34) [70–102, 139], 10 studies were adolescent patients (12 to 17 year olds), and 23 involved young adults (18 to 29 year olds); 31 of the 34 cases reported vaping THC or THC use was confirmed through urine analysis. Different diagnostic presentations of individuals with EVALI were provided in the articles, which aligned with the broad case definition established by the CDC, which includes (1) e-cigarette use or dabbing resin within 90 days of symptom onset, (2) pulmonary infiltrate present on radiographs, (3) no evidence of pulmonary infection with minimum workup of a viral respiratory panel and influenza testing, and (4) no evidence of alternative plausible diagnosis [4]. Studies highlighted a need to assess history of vaping use behaviors for patients with EVALI-like symptoms.

The case series papers (*n* = 25) provided insight into patterns of and risk factors for EVALI across individual patients [103–128, 137]. For example, among 160 patients reported to the California Department of Public Health, the median age of participants was 27 years, 62% were male, and 46% of patients were admitted to the intensive care unit [113]. Among the vaping devices gathered from participants (87 devices from 24 patients), 56% contained THC, and vitamin E acetate was found in 84% of the THC vape products [113]. Another case series of EVALI patients (*n* = 86) in Wisconsin and Illinois added to these findings by identifying Dank Vapes as a potential source of the contaminated cartridges; 66% of patients reported use of Dank Vapes [118]. In addition to risk factors for EVALI, case series such as Kalininsky et al. described treatment considerations and recommendations [106]. Treatment recommendations for EVALI included (1) supportive respiratory support as needed (i.e., supplemental oxygen, ventilation), (2) antibiotics for severe cases, per blood culture results, (3) antivirals until influenza is excluded (if symptoms occur during flu season), and (4) corticosteroids to treat lung inflammation.

In the cross-sectional studies (*n* = 9), [126, 129•, 130–136] research highlighted demographic characteristics of EVALI patients, substance use behaviors, and risk factors for EVALI. Common risk factors across studies for EVALI included younger age, cannabis vaping, higher frequency of cannabis vaping, the use of Dank Vapes (a brand of e-cigarettes only available on the black market), as well as obtaining products from informal sources. These findings are supported by current evidence from the CDC; as of the last update (data from January 14, 2020) [140], 76% of EVALI patients were under 35 years of age. Blount et al. provided the strongest evidence on the link between vitamin E acetate and development of EVALI; vitamin E acetate was found in 94% of patients’ BAL (bronchoalveolar-lavage) fluid and was in BAL fluid taken from the control participants [130].

#### Other Health Issues

About 24.7% (*n* = 23) of the studies categorized under this sub-topic examined other health effects of cannabis vaping, including oral health issues, other respiratory effects, cardiotoxicity, and seizures [65, 141–162]. Young persons who vape cannabis with oral health issues presented with conditions such as dental caries, erosions, and ulcerations [147]. Case reports of teens who vape cannabis described someone with daily use presenting with seizures [149] and someone who vaped for the first time presented with myocardial ischemia following chest pain [150]. One young who vaped cannabis who presented with respiratory issues had bronchiolitis (with mechanism distinct from EVALI) [152]; while two cases who vaped cannabis were treated for nontuberculous mycobacteria (NTM) infection [153]. Another case report of a young person who vaped cannabis oil described symptoms of catatonia, mania, and psychosis supposedly induced by previous cannabis vaping [151]. Cases of eosinophilic pneumonitis, synthetic cannabinoid intoxication, Torsades de pointes, and cardiac arrest were reported among youth who vaped various forms of marijuana [155, 160–162]. A qualitative study which interviewed cannabis vaping youth reported participants describing the physical health effects ranging from respiratory, oral, nausea, and appetite related and headaches [157].

Other observational studies examined subjective effects, functioning (physical, social, and mental), and respiratory effects such as bronchitis and wheezing [65, 144–146, 156]. An intensive longitudinal study on subjective cannabis intoxication and modes of administration found that participants reported higher intoxication on the days they used only bongs than the days they used only vapes [158].

Experimental studies examined the effects of vaporized cannabis on cognition, psychomotor performance, and physiological pain [141–143]. In a double-blind crossover study, Wilsey et al. examined analgesic efficacy of vaporized cannabis by comparing medium dose, low dose, and placebo treatment [142]. Subjects reported greater pain relief (measured by visual analog scale and Patient Global Impression of Change) by using cannabis compared to placebo, while results were almost similar for low and high dose. Spindle et al. examined acute dose effects of smoked and vaporized cannabis in a double-blind, crossover study [141]. They reported higher peak concentrations of THC in blood and stronger drug effects for pharmacodynamic outcomes (subjective drug effects, cognitive and psychomotor performance) by using vaporized cannabis as compared to equal doses of smoked cannabis. In another double-blind, crossover study, Arkell et al. examined the effects of vaporized THC-dominant cannabis (containing 11% THC and < 1% CBD), THC/CBD equivalent cannabis (containing 11% THC and 11% CBD), and placebo (containing < 1% THC/CBD) on driving and cognitive performance [143]. Compared to placebo, cognitive performance was impaired by both cannabis types, especially by the THC/CBD equivalent. Both cannabis types had little effect on driving performance, except for a car-following task, where increased lane weaving was reported for both types of cannabis. The authors conclude that adverse effects specific to cognitive and driving performance were similar for THC-dominant cannabis and THC/CBD equivalent cannabis and, in some cases, CBD may worsen THC-induced impairment. Another placebo-controlled randomized trial reported enhanced verbal episodic memory performance among those administered cannabidiol as compared to the placebo, while also indicating that CBD may not impact attention or working memory performance negatively as no significant effects were found for those outcomes [159]. Health effects associated with cannabis vaping among youth and young adults are summarized in Table 2.

**Table 2:** Health effects, risk factors and reasons associated with cannabis vaping among youth and young adults

Health effects of cannabis vaping: • Symptoms (bronchiolitis, bronchitis, wheezing, nontuberculous mycobacteria infection) • Respiratory gastrointestinal symptoms • Constitutional symptoms (cough, difficulty breathing, nausea, diarrhea, fever, chills, and/or weight loss) • Oral health issues (dental caries, erosions, ulcerations) • Myocardial ischemia • Mental health (psychosis, mania, catatonia) • Pain relief • Impaired cognitive performance
Risk factors or correlates of cannabis vaping: • Male sex • Older age (older youth or young adults relative to older adults) • Hispanic and Black race/ethnicity compared to White • Greater socio-economic status, greater parental education level • E-cigarette use with nicotine or flavors • E-cigarette use for reason trendy/cool • E-cigarette marketing exposure • Low disapproval of nicotine vaping • Other cannabis use (spliffs, blunts, pipes, bidis) • Medical cannabis use • Vaping cannabis in a vehicle • Obtaining cannabis from dispensaries or recreational retailers versus friend and family • Susceptibility to cannabis use • Low disapproval of smoking cannabis regularly • Other tobacco use (cigarette, cigars, hookah, smokeless tobacco) • Alcohol use or binge drinking • Non-medical use of prescription stimulant use or prescription opioids • Other illicit drug use • Easy access to e-cigarettes and cannabis • Lower perceived addictiveness of e-cigarettes • Lower perceived risk of cannabis use • Living with peers, parents, siblings who use cannabis • Endorsement by peers • Presence in social network • School-related factors (urban or suburban, low grade point average, skipped class, delinquent behavior) • Sensation seeking • Presence among social networks • Greater impulsivity and inattention (ADHD impulsivity) • Negative/positive urgency • Lack of perseverance • Openness to new experiences • Psychiatric symptoms (conduct problems, depressive symptoms, anhedonia, psychotic experiences) • Canadian immigration status Reasons for cannabis vaping: • To get high • Safer than smoking, combustion • Healthier than smoking • Less discomfort/irritation compared with smoking • Reduce or quit cigarette smoking, other combustible tobacco, or cannabis • Control dosage • Maintain sustained high • Experimentation • Mixing with flavors • Friend use • Looks trendy/cool • Enjoyment • Stress relief • Relaxation • Sleep improvement • Mood improvement • Efficient • Discreetness • Convenience • Circumvent smoking bans • Cheaper than smoked cannabis • Easily concealed

### Etiology

This review identified 48 studies that reported on the etiology of cannabis vaping [3, 21, 24, 28, 29, 31, 33, 43, 47, 50, 64, 163–196, 197•, 198–200]; most of these studies were cross-sectional ones (Table 1). The most commonly studied correlates were biological sex; age or grade level; race/ethnicity; socio-economic status (SES); other tobacco use, including nicotine vaping (i.e., e-cigarette use); and other modes of cannabis use (e.g., smoking). Consistent findings are summarized here. Across studies, cannabis vaping was more common among males than females [21, 24, 28, 29, 47, 170, 173, 178, 179, 182, 185, 198, 200], and among older youth (e.g., high school compared to middle school students) in most studies [21, 29, 47, 163, 167, 198], and among young adults compared to older adults [31, 50]. Cannabis vaping was also more common among Hispanic youth compared with non-Hispanic White [28, 163, 167, 190] or Black [21, 28, 167, 190, 198] youth. Some studies showed that Black youths were more likely to vape cannabis relative to White youth [64, 163], while other studies showed the reverse [21, 24, 198, 200]. Studies showed greater socio-economic status (SES) may be a risk factor for cannabis vaping [24, 47, 64, 170, 182]. E-cigarette use with nicotine only or flavors [28, 29, 163, 166, 172–174, 180, 186, 197•, 198, 200], as well as use of cigarettes, cigars, hookah, and smokeless tobacco [21, 28, 29, 50, 163, 166, 174, 178, 179, 186, 195, 197•, 199] is associated with greater odds of cannabis vaping. Former cannabis use, susceptibility to use cannabis, and peer use of cannabis, via vaping and other forms of cannabis consumption (e.g., ingesting, smoking), have also been shown to be related to greater odds of cannabis vaping [21, 169, 170, 173, 175, 179, 180, 184, 200]. Greater access to both e-cigarettes [33, 167, 197•] and cannabis [167]; and parent, sibling, and peer cannabis vaping [28, 64, 166, 180, 197•] are associated with cannabis vaping.

Other less studied correlates of cannabis vaping included alcohol use [170, 174, 197•, 198, 200], non-medical prescription use [174], and illicit drug use [170, 188]; greater impulsivity [174, 179] and attention-related factors (e.g., inattention, perseverance) [174]; openness to new experiences [182] and sensation seeking [170, 189]; psychiatric symptoms (e.g., depressive symptoms, conduct problems) [174, 182]; delinquent behavior [196]; low grade point average and skipping class [167]; internalizing and externalizing problems [197•, 198]; more exposure to e-cigarette marketing (see “Marketing” section, below) [179]; school urbanicity (urban and suburban being greater risk versus rural) [24, 198]; using cannabis in a vehicle [170]; obtaining cannabis from dispensaries or recreational retailers versus from friends and family [171]; low disapproval of nicotine vaping [167] and of smoking cannabis regularly, [182] lower perceived risk of cannabis use [167]; lower perceived addictiveness of e-cigarettes [163]; and earlier age of initiation of cannabis use, in any form [170, 200].

Seven studies reported on youth and young adults’ reasons for cannabis vaping [50, 178, 181–183, 187, 193]. Reasons included that vaping cannabis is safer, healthier, and/or less physically irritating than cigarettes or combustible tobacco; to reduce or quit smoking cigarettes or other combustible tobacco products, as well as cannabis; to control dosage or amount of cannabis consumed and/or to maintain a sustained high; experimentation; mixing with flavors; friends’ use; because cannabis vaping is “trendy” and “cool”; because it is enjoyable; stress relief; relaxation; sleep improvement; mood improvement; and its discreetness or ability to circumvent smoking bans. One study reported a reason that may deter young adults from cannabis vaping, namely that vaping may be less safe than other forms of consuming cannabis [181].

Four studies reported on youth and young adults’ harm perceptions of cannabis vaping [176, 177, 181, 191]. One study reported that young adults perceived combustible (e.g., smoked, via a joint or pipe) cannabis as more harmful than vaporized cannabis; however, chemicals (e.g., butane) used make cannabis concentrates were thought to be a source of harm [181]. Another study reported that most participants perceived using e-cigarettes to vape cannabis to be just as harmful or more harmful than joint use. Studies also reported uncertainty about the safety and harms of cannabis vaping compared to other forms of use [177, 191]. One study reported that youth and young adult cannabis vapers had lower odds of perceiving harm from daily cannabis use in any form, when compared to never cannabis users [176]. Risk factors and reasons for use associated with cannabis vaping among youth and young adults are summarized in Table 2.

### Marketing

A growing body of literature shows e-cigarette marketing is prevalent, largely unregulated, and is an important risk factor for e-cigarette use among youth and young adults, especially [201]. This review identified 11 studies that examined marketing and/or other types of messaging about using e-cigarettes and the relation to vaping cannabis-related substances. These studies primarily focused on digital media (i.e., internet and social media), which is highly utilized by e-cigarette manufacturers and other promoters due to lax regulations, low cost, and access to vast and diverse young audiences.

Most studies (*n* = 10) were descriptive content analyses of messaging about vaping cannabis on varied digital media platforms including e-liquid vendor online sites [202], the Google Play Store [203], YouTube [204, 205], Instagram [206–209], and Twitter [210, 211]. Together, these studies showed that content was primarily promotional, with very little prevention, cessation, or health messaging. In 2020, in an effort to combat COVID-19, South Africa restricted tobacco and e-cigarette product sales over a 5-month period. Studying this, Agaku et al. showed that among 2661 e-liquids marketed by online vendors, about 29% were cannabidiol (CBD) liquids, primarily fruit and tobacco-flavored [202]. During the restriction period, online vendors commonly promoted CBD liquids instead of nicotine-containing e-liquid, salts, and concentrates [202]. Meacham et al. identified 79 Google Play Store applications that were related to vaping, three of which were specific to cannabis vaping, with the remaining specific to nicotine or unspecified [203]. Applications were commonly categorized as “tools and lifestyle,” “health and fitness,” and “social” and included do-it-yourself content for creating e-liquids and coils, games, social networking, purchasing e-cigarette products, smoking cessation services, pairing with e-cigarettes to adjust dosage and temperature, and e-cigarette cessation [203]. Yang et al. identified 214 YouTube videos over a 1-year period in 2014–2015 related to cannabis vaping [204]. Most videos were generated and shared by lay persons and included personal experiences and tips, instructions, and product reviews related to cannabis vaping — although 21% of videos were clearly marketing a specific brand or product [204]. Over a 4-month period in 2018, Ramamurthi et al. identified 18,200 “stealth vaping” YouTube videos that touted numerous discreetly designed e-cigarettes like JUUL’s USB-like device, to enable discreet vaping of nicotine and cannabis, especially by youth [205]. E-cigarettes in these videos resembled pens, smart phones and other electronics, and even asthma inhalers [205]. Over a 2-week period in 2014, Cavazos-Rehg et al. identified over 400,000 Instagram posts with cannabis-related hashtags [206]. Of a random sample of 5000 of these posts, 2136 were explicitly about cannabis (generally) [206]. About 9% (187/2136) of cannabis-related posts were advertisements, many of which (43%; 80/187) promoted devices or tools to use cannabis including advertising vape pens to use cannabis (13%; 10/80) [206]. Majmundar et al. identified 1775 Instagram posts with the hashtag #kandypens posted over a 1-month period in 2018, and about 32% of these posts referenced using cannabis-related solutions in Kandy Pens [207]. These posts were made and distributed by laypersons, vendors, KandyPen’s official Instagram account, vaping advocates or enthusiasts, and influencers [207]. Czaplicki et al. identified over 50,000 JUUL-related Instagram posts over 2 ½ months in 2018, prior to JUUL Lab’s voluntary actions limiting their own youth-oriented Instagram content in May 2018, and over 6 months following these self-imposed restrictions in 2018 [208]. Cannabis-content in JUUL-related posts was identified as a common theme, and one that grew in prominence over time in 2018 [208]. Kostygina et al. identified cannabis vaping themes in JUUL-related Instagram posts over 2 ½ months in 2018. Cross-promotion of JUUL with cannabis products was a prominent theme in commercial posts, and youth use of JUUL with marijuana was a prominent theme in organic, non-commercial posts [209]. Two studies of e-cigarette-related tweets posted in 2019 identified cannabis vaping as a prominent theme, for example, cannabis vaping was discussed on Twitter as “the real problem” causing the EVALI outbreak [210, 211].

Only one study reported statistically significant longitudinal associations between marketing via numerous channels including digital media and cannabis vaping [179]. Kreitzberg et al. reported increased odds of using e-cigarette products with cannabis 1-year later among 3720 college students who self-reported increased exposure to e-cigarette advertising via eight channels: gas/convenience stores, drug stores, grocery stores; liquor stores; bars or clubs; music events; radio or internet radio; online; magazines or newspapers; and billboards [179].

### Regulation

As of May 2021, a total of 36 states and 4 US territories allow for medical use of cannabis products and 18 states, 2 US territories, and the District of Columbia have legalized adult-use (recreational) cannabis [212]. Of note, two states (South Dakota and Mississippi) passed ballot initiatives by popular vote to legalize cannabis use (medical and adult-use in South Dakota; medical-only in Mississippi) in 2020; however, these ballot initiatives were overturned by the State Supreme Court in each state [212]. Most states set no legal age restrictions for medical cannabis, though all require a prescription from a credentialed medical professional and for minors to use under the supervision of their guardian. Legal age for purchase of adult-use cannabis is 21 years of age in all legalized states, territories, and the District of Columbia [212].

Several articles explored cannabis vaping prevalence among youth and young adults by state-level cannabis regulatory policy [213•, 214–219]. Across the USA, studies show the prevalence of any form of cannabis consumption among young people, including for cannabis vaping, is consistently higher in legalized states, relative to prohibition states [213•, 220, 221]. One study also showed that among youth who had ever used cannabis, those living in a legalized state initiated cannabis vaping at a slightly earlier age than those in prohibition states (mean age = 15.3 vs. 15.4 years; *p* < 0.01) [42]. Another study showed that cannabis vaping (and other forms of cannabis consumption) was more prevalent among young adults compared to older adults in states where recreational cannabis use is legal [62]. Canada legalized recreational marijuana use for adults in 2018. One study reinforced that legalization does not appear to strengthen barriers to obtaining cannabis for underage youth [55]. Increases in multimodal use of cannabis (e.g., smoking, vaping, and/or ingesting) among underage youth were observed after legislation was passed [54, 56].

Readers are referred to a summary [35] that describes the regulatory and marketing environment related to legalization of adult-use cannabis in 11 legalized states. This paper noted that regulatory approaches vary substantially but most states set standards on (1) quantity; (2) eligibility for purchase; (3) transportation; (4) retail setting; and (5) how cannabis can be grown, packaged, sold, distributed, and promoted. Regulatory approaches include rules and guidelines for cannabis vaping products, including establishing quantity limits for THC concentrates as well as requiring labels to state the same and whether concentrates have been tested for contaminants. Strong enforcement efforts are necessary, as studies report that many underage youth and young adults successfully purchase their devices online or from vape shops [222].

## Discussion

From 2013 forward, consistent with increases in cannabis vaping among young people [2•], the available research literature on this topic has grown exponentially. We hope this review provides a timely summary of the state-of-the-knowledge, in ways that inform development of preventive interventions and provides direction for future studies. Clearly, surveillance of cannabis vaping among young people should continue. It would be beneficial for efforts to continue to collect information that informs comparisons by substance (e.g., THC, CBD vs. nicotine) and method (e.g., vaping vs. smoking) of use, so that other streams of research (e.g., on health effects) can anchor appropriately into this information. Although reports of EVALI have declined over time, a body of literature is starting to emerge regarding the other health effects of cannabis vaping. To date, little research has examined its impact on gastrointestinal issues, despite a larger body of evidence on the same for cannabis smoking [65, 141–153]. More studies of the impact of cannabis vaping on cognition, affect, and behavior will also be useful, given limited research to date. Preventive interventions can benefit from the information summarized here, which highlights important target audiences that can benefit given their higher prevalence of use (e.g., older youth, young adults; Hispanic youth) and risk factors (e.g., reasons for use, risk perceptions) that could be relevant to the development of program or communication campaign content.

Overall, there is a dearth of research on marketing of and/or other types of messaging about cannabis vaping. More research is needed to describe the various channels through which cannabis vaping is promoted especially via the ever-expanding world of digital media, as well as the content and source of messages about cannabis vaping. Although Apple, Facebook, and many popular, digital media companies have self-imposed policies against tobacco, e-cigarette, cannabis, and other drug use, some studies have shown these are lacking in preventing youth and young adults’ exposure to tobacco and other substance use-related content via these media [223]. Additionally, there are no federal restrictions on digital marketing to curb commercial e-cigarette marketing, at least. The nine studies identified in this review suggest e-cigarette — and particularly cannabis vaping — marketing and other promotional messaging appear to be critical factors contributing to the explosion of cannabis vaping among youth and young adults.

## Conclusions

As the regulatory environment for cannabis products continues to open up not only across many states in the USA but also in other countries like Uruguay, Canada, Mexico, and South Africa, the need for research and intervention specific to youth and young adults will become increasingly relevant. The research to-date clearly shows that cannabis vaping among young people is consistently higher in settings where use is legal for those over the age of 21 years. Therefore, additional methods for preventive interventions will be necessary in the future, and development of these must be informed by etiologic research. Already, as evidenced by EVALI, the large majority of the negative impact of cannabis vaping on health has been disproportionately experienced by young people. They will continue to require support from the scientific community in the future, in order to mitigate any additional damage to their well-being, collectively, that lies ahead.

## Supplementary Information

Below is the link to the electronic supplementary material.

Supplementary file1 (DOCX 30 KB)

Supplementary file2 (DOCX 39 KB)

Supplementary file3 (DOCX 39 KB)

Supplementary file4 (DOCX 22 KB)

Supplementary file5 (DOCX 53 KB)
