# Supplementary figures and images for: Cannabis Vaping Among Youth and Young Adults: a Scoping Review
Source: Curr Addict Rep. 2022 May 7;9(3):217–34. doi: 10.1007/s40429-022-00413-y (PMC9078633; doi:10.1007/s40429-022-00413-y)

**Figure 1: Cannabis vaping studies yearly publication trend**
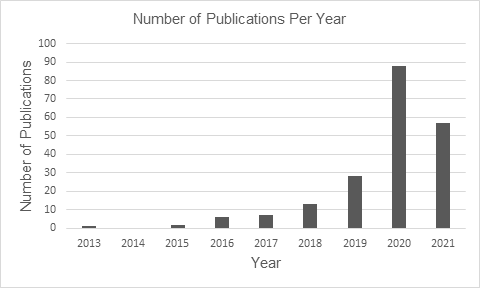

Supplement: Supplementary file 1 — Supplementary file1 (DOCX 30 KB) [file 40429_2022_413_MOESM1_ESM.docx]
