# Supplementary material for: Cannabis Vaping Among Youth and Young Adults: a Scoping Review
Source: Curr Addict Rep. 2022 May 7;9(3):217–34. doi: 10.1007/s40429-022-00413-y (PMC9078633; doi:10.1007/s40429-022-00413-y)
Supplement: Supplementary file 2 — Supplementary file2 (DOCX 39 KB) [file 40429_2022_413_MOESM2_ESM.docx]

**Supplementary Figure 2. Trends in lifetime (i.e., ever) cannabis vaping, Monitoring the Future (2017-2021)**

Source (youth): <http://monitoringthefuture.org/pubs/monographs/mtf-overview2021.pdf> (Table 1)

Prevalence of lifetime (i.e., ever) cannabis vaping

Source (young adults): <http://www.monitoringthefuture.org/pubs/monographs/mtf-vol2_2020.pdf> (Table 9-1)
