# Supplementary material for: Cannabis Vaping Among Youth and Young Adults: a Scoping Review
Source: Curr Addict Rep. 2022 May 7;9(3):217–34. doi: 10.1007/s40429-022-00413-y (PMC9078633; doi:10.1007/s40429-022-00413-y)
Supplement: Supplementary file 3 — Supplementary file3 (DOCX 39 KB) [file 40429_2022_413_MOESM3_ESM.docx]

**Supplementary Figure 3. Trends in past 30-day cannabis vaping, Monitoring the Future (2017-2021)**

Source (young adults): <http://www.monitoringthefuture.org/pubs/monographs/mtf-vol2_2020.pdf> (Table 9-3)

Source (youth): http://monitoringthefuture.org/pubs/monographs/mtf-overview2021.pdf (Table 3)

Prevalence of past 30-day cannabis vaping
