# Supplementary material for: Cannabis Vaping Among Youth and Young Adults: a Scoping Review
Source: Curr Addict Rep. 2022 May 7;9(3):217–34. doi: 10.1007/s40429-022-00413-y (PMC9078633; doi:10.1007/s40429-022-00413-y)
Supplement: Supplementary file 4 — Supplementary file4 (DOCX 22 KB) [file 40429_2022_413_MOESM4_ESM.docx]

| **Supplementary Table 1: Database search strategies** |
| --- |
| **PubMed**  ("electronic nicotine delivery systems"[Mesh] OR “vaping"[Mesh] OR “JUUL*” OR “ENDS”)  AND  ("cannabis"[Mesh] OR "marijuana smoking"[Mesh] OR "marijuana abuse"[Mesh] OR "marijuana use"[Mesh] OR “medical marijuana"[Mesh] OR “dronabinol” [Mesh] OR “cannabinoids” [Mesh] OR “CBD”)  AND  ("adolescent"[Mesh] OR "young adult"[Mesh] OR "minors" [Mesh] OR "students" [Mesh] OR "child" [Mesh] OR “college student*”)  AND  (2007:2021[pdat]) |
| **Embase**  ('electronic cigarette'/exp OR 'vaping'/exp OR 'JUUL'/exp OR 'ENDS'/exp)  AND  ('cannabis'/exp OR ‘medicinal cannabis’/exp OR ‘cannabis smoking’/exp OR ‘cannabis addiction’/exp OR ‘cannabis use’/exp OR ‘dronabinol’/exp OR ‘tetrahydrocannabinol’/exp OR ‘cannabinoid’/exp OR “CBD”)  AND  ('adolescent'/exp OR 'child'/exp OR ‘minor (person)’/exp OR 'student'/exp OR 'young adult'/exp OR ‘juvenile’/exp)  AND  [2007-2021]/py  AND  [embase]/lim |
| **Web of Science**  TS=('electronic cigarette* OR ‘e-cig*’ OR 'vaping*' OR ‘vape*’ OR 'electronic nicotine delivery system*' OR 'JUUL*' OR ‘ENDS’)  AND  TS=('cannabis’ OR 'marijuana’ OR 'cannabinoid' OR ‘cannabidiol’ OR 'Tetrahydrocannabinol' OR 'THC’ or ‘CBD’)  AND  TS=(‘adolescent*’ OR ‘child*’ OR ‘minor*’ OR ‘student*’ OR ‘young adult*’ OR ‘juvenile*’ OR ‘student*’ OR ‘college student*’ OR ‘teen*’ OR ‘youth*’)  AND  PY=(2007-2021) |
