# Supplementary material for: Cannabis Vaping Among Youth and Young Adults: a Scoping Review
Source: Curr Addict Rep. 2022 May 7;9(3):217–34. doi: 10.1007/s40429-022-00413-y (PMC9078633; doi:10.1007/s40429-022-00413-y)
Supplement: Supplementary file 5 — Supplementary file5 (DOCX 53 KB) [file 40429_2022_413_MOESM5_ESM.docx]

| **Supplementary Table 2. List of 202 studies included in scoping review** | | |
| --- | --- | --- |
| **Year** | **First Author** | **Title** |
| 2013 | Wilsey | Low-dose vaporized cannabis significantly improves neuropathic pain |
| 2015 | Hasin | Medical marijuana laws and adolescent marijuana use in the USA from 1991 to 2014: Results from annual, repeated cross-sectional surveys. |
| 2015 | Morean | High school students' use of electronic cigarettes to vaporize cannabis |
| 2016 | Cavazos-Rehg | Marijuana-related posts on Instagram |
| 2016 | Hakkarainen | Vaporizing the pot world – easy, healthy, and cool |
| 2016 | Jones | Prevalence and correlates of vaping cannabis in a sample of young adults |
| 2016 | Keyes | How does state marijuana policy affect us youth? Medical marijuana laws, marijuana use and perceived harmfulness: 1991–2014 |
| 2016 | Mammen | Vaporizing cannabis through e-cigarettes: Prevalence and socio-demographic correlates among Ontario high school students |
| 2016 | McDonald | Traversing the triangulum: the intersection of tobacco, legalised marijuana and electronic vaporisers in Denver, Colorado |
| 2017 | Borodovsky | U.S. cannabis legalization and use of vaping and edible products among youth |
| 2017 | Eggers | Youth use of electronic vapor products and blunts for administering cannabis |
| 2017 | Kenne | The use of substances other than nicotine in electronic cigarettes among college students |
| 2017 | Mehta | Myocardial ischemia with cannabinoid use in an adolescent |
| 2017 | Popova | Perceived harms and benefits of tobacco, marijuana, and electronic vaporizers among young adults in Colorado: Implications for health education and research |
| 2017 | Rickner | A little "dab" will do ya' in: A case report of neuro-and cardiotoxicity following use of cannabis concentrates |
| 2017 | Thurtle | Prevalence of use of electronic nicotine delivery systems (ENDS) to vape recreational drugs by club patrons in south London |
| 2018 | Cassidy | Initiation of vaporizing cannabis: individual and social network predictors in a longitudinal study of young adults |
| 2018 | Dilley | Prevalence of cannabis use in youths after legalization in Washington state. |
| 2018 | Frohe | Correlates of cannabis vape-pen use and knowledge among U.S. college students |
| 2018 | Hoffenberg | Marijuana use by adolescents and young adults with inflammatory bowel disease |
| 2018 | Jones | Comparison of the locations where young adults smoke, vape, and eat/drink cannabis: Implications for harm reduction |
| 2018 | Kerr | Changes in marijuana use across the 2012 Washington state recreational legalization: Is retrospective assessment of use before legalization more accurate? |
| 2018 | Peters | Prevalence and sociodemographic correlates of adolescent use and polyuse of combustible, vaporized, and edible cannabis products |
| 2018 | Sarvet | Medical marijuana laws and adolescent marijuana use in the United States: A systematic review and meta‐analysis. |
| 2018 | Spindle | Acute effects of smoked and vaporized cannabis in healthy adults who infrequently use cannabis: A crossover trial |
| 2018 | Steigerwald | Smoking, vaping, and use of edibles and other forms of marijuana among U.S. adults |
| 2018 | Tam | Students’ cigarette smoking and the perceived nicotine content of their e-cigarettes |
| 2018 | Trivers | Prevalence of cannabis use in electronic cigarettes among us youth |
| 2018 | Yang | How is marijuana vaping portrayed on YouTube? Content, features, popularity and retransmission of vaping marijuana YouTube videos |
| 2019 | Arkell | Cannabidiol (CBD) content in vaporized cannabis does not prevent tetrahydrocannabinol (THC)-induced impairment of driving and cognition |
| 2019 | Blagev | Clinical presentation, treatment, and short-term outcomes of lung injury associated with e-cigarettes or vaping: A prospective observational cohort study |
| 2019 | Cerdá | Association between recreational marijuana legalization in the United States and changes in marijuana use and cannabis use disorder from 2008 to 2016. |
| 2019 | Fataar | The prevalence of vaping and smoking as modes of delivery for nicotine and cannabis among youth in Canada, England and the United States |
| 2019 | Gaub | Patient characteristics and product use behaviors among persons with e-cigarette, or vaping, product use-associated lung injury - Indiana, June – October 2019 |
| 2019 | Ghinai | Inpatient notes: Clinical pearls-e-cigarette, or vaping, product use-associated lung injury |
| 2019 | Ghinai | E-cigarette product use, or vaping, among persons with associated lung injury - Illinois and Wisconsin, April-September 2019 |
| 2019 | Hall | Public health implications of legalising the production and sale of cannabis for medicinal and recreational use. |
| 2019 | Jackson | It's all the rage! Exploring the nuances in the link between vaping and adolescent delinquency |
| 2019 | Kalininskiy | E-cigarette, or vaping, product use associated lung injury (EVALI): Case series and diagnostic approach |
| 2019 | Knapp | Emerging trends in cannabis administration among adolescent cannabis users |
| 2019 | Kotlaja | Cannabis prevalence and national drug policy in 27 countries: An analysis of adolescent substance use |
| 2019 | Kowitt | Vaping cannabis among adolescents: prevalence and associations with tobacco use from a cross-sectional study in the USA |
| 2019 | Kreitzberg | Exposure to ends advertising and use of marijuana in ends among college students |
| 2019 | Landman | Life-threatening bronchiolitis related to electronic cigarette use in a Canadian youth |
| 2019 | Lewis | E-cigarette use, or vaping, practices and characteristics among persons with associated lung injury - Utah, April-October 2019 |
| 2019 | Merianos | Characteristics of daily e-cigarette use and acquisition means among a national sample of adolescents |
| 2019 | Miech | The national prevalence of adolescent nicotine use in 2017: Estimates taking into account student reports of substances vaped |
| 2019 | Moritz | Update: characteristics of patients in a national outbreak of e-cigarette, or vaping, product use-associated lung injuries - United States, October 2019 |
| 2019 | Navon | Risk factors for e-cigarette, or vaping, product use-associated lung injury (EVALI) among adults who use e-cigarette, or vaping, products - Illinois, July - October 2019 |
| 2019 | Nguyen | Past 30-day co-use of tobacco and marijuana products among adolescents and young adults in California |
| 2019 | Perrine | Characteristics of a multistate outbreak of lung injury associated with e-cigarette use, or vaping - United States, 2019 |
| 2019 | Ramamurthi | Juul and other stealth vaporisers: hiding the habit from parents and teachers |
| 2019 | Schneider | Patterns of co-occurring modes of marijuana use among Colorado high school students |
| 2019 | Smart | Early evidence of the impact of cannabis legalization on cannabis use, cannabis use disorder, and the use of other substances: findings from state policy evaluations |
| 2019 | Taylor | Characteristics of e-cigarette, or vaping, products used by patients with associated lung injury and products seized by law enforcement - Minnesota, 2018 and 2019 |
| 2019 | Tucker | Types of cannabis and tobacco/nicotine co-use and associated outcomes in young adulthood |
| 2019 | Yoo | Perceptions of the comparative safety of different forms of marijuana use among the adult us population |
| 2020 | Abeles | Vaping-associated lung injury caused by inhalation of cannabis oil |
| 2020 | Aberegg | Diagnosis of EVALI: General approach and the role of bronchoscopy |
| 2020 | Adkins | Demographics, substance use behaviors, and clinical characteristics of adolescents with e-cigarette, or vaping, product use-associated lung injury (EVALI) in the United States in 2019 |
| 2020 | Aldy | Severe e-cigarette, or vaping, product use associated lung injury requiring venovenous extracorporeal membrane oxygenation |
| 2020 | Ali | Oral erosions associated with surreptitious marijuana vaping in an adolescent boy |
| 2020 | Amin | Do not huff, puff, or vape that stuff: Interstitial airspace disease in a teenager |
| 2020 | Antwi-Amoabeng | Vaping is not safe: A case of acute eosinophilic pneumonia following cannabis vapor inhalation |
| 2020 | Artunduaga | Pediatric chest radiographic and CT findings of electronic cigarette or vaping product use-associated lung injury (EVALI) |
| 2020 | Barrington-Trimis | Characterizing the predictive validity of measures of susceptibility to future use of combustible, vaporized and edible cannabis products in adolescent never-users |
| 2020 | Barrington-Trimis | Risk of persistence and progression of use of 5 cannabis products after experimentation among adolescents |
| 2020 | Ben | Vaping while high: Factors associated with vaping marijuana among youth in the United States |
| 2020 | Blount | Vitamin E acetate in bronchoalveolar-lavage fluid associated with EVALI |
| 2020 | Boisvert | Subjective effects of combustible, vaporized, and edible cannabis: Results from a survey of adolescent cannabis users |
| 2020 | Braymiller | Assessment of nicotine and cannabis vaping and respiratory symptoms in young adults |
| 2020 | Campbell | Correlates of lifetime blunt/spliff use among cigarette smokers in substance use disorders treatment |
| 2020 | Chidambaram | Clinical and radiological characteristics of e-cigarette or vaping product use associated lung injury |
| 2020 | Clawson | Profiles of nicotine and cannabis exposure among young adults with asthma |
| 2020 | Conuel | Cannabinoid oil vaping-associated lung injury and its radiographic appearance |
| 2020 | Corcoran | The importance of anti-vaping vigilance-EVALI in seven adolescent pediatric patients in Northeast Ohio |
| 2020 | Czaplicki | #Toolittletoolate: JUUL-related content on Instagram before and after self-regulatory action |
| 2020 | D'Amico | Sources of cannabis among young adults and associations with cannabis-related outcomes |
| 2020 | Dai | Self-reported marijuana use in electronic cigarettes among us youth, 2017 to 2018 |
| 2020 | Dai | Use of e-cigarettes for nicotine, marijuana, and just flavoring among U.S. youth |
| 2020 | Darmawan | Vaping in today’s pandemic: E-cigarette, or vaping, product use-associated lung injury mimicking COVID-19 in teenagers presenting with respiratory distress |
| 2020 | Doukas | E-cigarette or vaping induced lung injury: A case series and literature review |
| 2020 | Dugas | Type of e-liquid vaped, poly-nicotine use and nicotine dependence symptoms in young adult e-cigarette users: A descriptive study |
| 2020 | Dunbar | Developmental trajectories of tobacco/nicotine and cannabis use and patterns of product co-use in young adulthood |
| 2020 | Ellington | Update: Product, substance-use, and demographic characteristics of hospitalized patients in a nationwide outbreak of e-cigarette, or vaping, product use-associated lung injury - United States, August 2019-January 2020 |
| 2020 | Espinosa | 19-year-old man with fevers, abdominal pain, and cough |
| 2020 | Fedt | Vaping-associated lung injury: A new cause of acute respiratory failure |
| 2020 | Fryman | Acute respiratory failure associated with vaping |
| 2020 | Gaiha | Underage youth and young adult e-cigarette use and access before and during the coronavirus disease 2019 pandemic |
| 2020 | Hall | Assessing the public health impacts of legalizing recreational cannabis use: The US experience. |
| 2020 | Heinzerling | Severe lung injury associated with use of e-cigarette, or vaping, products-California, 2019 |
| 2020 | Hilton | E-cigarettes and vaping associated lung injury: A case series and brief review |
| 2020 | Hoffmann | Sensation seeking and adolescent e-cigarette use |
| 2020 | Irusa | Potential oral health effects of e-cigarettes and vaping: A review and case reports |
| 2020 | Israel | A unique case of secondary pulmonary alveolar proteinosis after e-cigarette, or vaping, product use-associated lung injury |
| 2020 | Kass | Case series: Adolescent victims of the vaping public health crisis with pulmonary complications |
| 2020 | Kastaun | Electronic cigarettes to vaporize cannabis: Prevalence of use and associated factors among current electronic cigarette users in Germany (DEBRA study) |
| 2020 | Kaur | Associations between digital technology and substance use among U.S. adolescents: Results from the 2018 monitoring the future survey |
| 2020 | Kelley | Vaping-associated lung injury: Should we consider screening adolescents who vape? |
| 2020 | Khiatah | Vaping-induced lung injury: Brief report for the practicing clinician |
| 2020 | Kolar | Routes of cannabis administration among adolescents during criminal prohibition of cannabis in Canada |
| 2020 | Kooragayalu | Vaping associated pulmonary injury (VAPI) with superimposed mycoplasma pneumoniae infection |
| 2020 | Krishnasamy | Update: characteristics of a nationwide outbreak of e-cigarette, or vaping, product use-associated lung injury - United States, august 2019-january 2020 |
| 2020 | Lanza | Trajectories of nicotine and cannabis vaping and polyuse from adolescence to young adulthood |
| 2020 | Layden | Pulmonary illness related to e-cigarette use in Illinois and Wisconsin - final report |
| 2020 | Lenk | Practices and policies of marijuana retail stores in the first two us states to legalize recreational marijuana sales |
| 2020 | Leos-Toro | Cannabis health knowledge and risk perceptions among Canadian youth and young adults |
| 2020 | Leventhal | Psychiatric comorbidity in adolescent use and poly-use of combustible, vaporized, and edible cannabis products |
| 2020 | MacMurdo | E-cigarette or vaping product use-associated lung injury: Clinical, radiologic, and pathologic findings of 15 cases |
| 2020 | Majmundar | Characterising KandyPens-related posts to Instagram: implications for nicotine and cannabis use |
| 2020 | Manning | Vaping cannabis oil: A case of catatonia associated with use of high-potency cannabis |
| 2020 | Meacham | Vaping-related mobile apps available in the google play store after the apple ban: Content review |
| 2020 | Messina | Cytotoxic lesion of the splenium of the corpus callosum in a patient with EVALI |
| 2020 | Miech | Trends in reported marijuana vaping among us adolescents, 2017-2019 |
| 2020 | Mull | Eosinophilic pneumonia and lymphadenopathy associated with vaping and tetrahydrocannabinol use |
| 2020 | Mull | Bronchiectasis associated with electronic cigarette use: A case series |
| 2020 | Nicksic | Cannabis legalization, tobacco prevention policies, and cannabis use in e-cigarettes among youth |
| 2020 | O'Carroll | Vaping-associated lung injury |
| 2020 | Ocampo-Gonzalez | Cytologic features of vaping-induced lung injury: A case report |
| 2020 | Odish | E-cigarette, or vaping, product use-associated lung injury (EVALI) treated with veno-venous extracorporeal membrane oxygenation (VV-ECMO) and ultra-protective ventilator settings |
| 2020 | Patrick | Trends in marijuana vaping and edible consumption from 2015 to 2018 among adolescents in the US |
| 2020 | Perrenoud | Vaping-induced lung injury in a 21-year-old woman |
| 2020 | Phung | Pediatric acute respiratory distress syndrome and hypersensitivity pneumonitis related to e-cigarette vaping |
| 2020 | Pokhrel | Predictors of marijuana vaping onset and escalation among young adults |
| 2020 | Pray | E-cigarette, or vaping, product use-associated lung injury among clusters of patients reporting shared product use - Wisconsin, 2019 |
| 2020 | Puebla | Discordant bilateral bronchoalveolar lavage findings in a patient with acute eosinophilic pneumonia associated with counterfeit tetrahydrocannabinol oil vaping |
| 2020 | Rao | Clinical features of e-cigarette, or vaping, product use-associated lung injury in teenagers |
| 2020 | Rasmussen | A case of vaping-associated acute lung injury |
| 2020 | Rodriguez | E-cigarette or vaping product use-associated lung injury (EVALI) mimicking covid-19 disease |
| 2020 | Ronald | Extensive pneumorrhachis associated with vaping-induced lung injury |
| 2020 | Sakuma | Hookah and electronic inhalant device use and perceptions among African American youth and young adults: Are we asking the right questions? |
| 2020 | Schauer | Modes of marijuana use - smoking, vaping, eating, and dabbing: Results from the 2016 BRFSS in 12 states |
| 2020 | Seaman | Use of tobacco products/devices for marijuana consumption and association with substance use problems among U.S. young adults (2015-2016) |
| 2020 | Shah | E-cigarette or vaping product use-associated lung injury: A case study and review of the literature |
| 2020 | Sharma | Electronic vaping product use among young adults who receive care at a major medical institution |
| 2020 | Silverman | Vaping induced pneumonitis: A small community hospital's case series and analysis |
| 2020 | Smith | A case of e-cigarette, or vaping, product use-associated lung injury (EVALI) in a previously healthy patient: Case report and literature review |
| 2020 | Tai | The future of substance abuse now: Relationships among adolescent use of vaping devices, marijuana, and synthetic cannabinoids |
| 2020 | Terry-McElrath | Changes In The Order Of Cigarette And Marijuana Initiation And Associations With Cigarette Use, Nicotine Vaping, And Marijuana Use: US 12th Grade Students, 2000-2019 |
| 2020 | Thakrar | E-cigarette, or vaping, product use-associated lung injury in adolescents: A review of imaging features |
| 2020 | Uddin | Prevalence, trends, and distribution of nicotine and marijuana use in e-cigarettes among us adults: The behavioral risk factor surveillance system 2016‚äì2018 |
| 2020 | Wang | E-cigarette or vaping product use-associated lung injury in the pediatric population: Imaging features at presentation and short-term follow-up |
| 2020 | Werner | Hospitalizations and deaths associated with EVALI |
| 2020 | Wheeler | CBD (cannabidiol) product attitudes, knowledge, and use among young adults |
| 2020 | Young-Wolff | Routes of cannabis administration among females in the year before and during pregnancy: Results from a pilot project |
| 2021 | Adhikari | EVALI - e-cigarette or vaping product use-associated lung injury: A case report |
| 2021 | Agaku | Circumvention of covid-19-related restrictions on tobacco sales by the e-cigarette industry in south Africa and comparative analyses of heated tobacco product vs combustible cigarette volume sales during 2018-2020 |
| 2021 | Ahmed | Vaping cannabis butane hash oil leads to severe acute respiratory distress syndrome – A case of EVALI in a teenager with hypertrophic cardiomyopathy |
| 2021 | Algiers | Content analysis of us newspaper coverage of causes and solutions to vaping-associated lung injury |
| 2021 | Ball | The rise of cannabis vaping: implications for survey design |
| 2021 | Boccio | Adolescent nicotine and marijuana vaping activity and the use of other illicit substances |
| 2021 | Boyd | Cannabis, vaping, and respiratory symptoms in a probability sample of us youth |
| 2021 | Chen | Vaping associated pulmonary injury (VAPI) with superimposed mycoplasma pneumoniae infection |
| 2021 | Clendennen | Understanding college students' experiences using e-cigarettes and marijuana through qualitative interviews |
| 2021 | Cloutier | Associations of mode of administration on cannabis consumption and subjective intoxication in daily life |
| 2021 | Cwalina | Prevalence of young adult vaping, substance vaped, and purchase location across five categories of vaping devices |
| 2021 | Doggett | Modes of cannabis use among Canadian youth in the compass study; using LCA to examine patterns of smoking, vaping, and eating/drinking cannabis |
| 2021 | Doggett | Patterns of cannabis use among Canadian youth over time; examining changes in mode and frequency using latent transition analysis |
| 2021 | Farsalinos | Changes from 2017 to 2018 in e-cigarette use and in ever marijuana use with e-cigarettes among us adolescents: Analysis of the national youth tobacco survey |
| 2021 | Fischer | An overview of select cannabis use and supply indicators pre- and post-legalization in Canada |
| 2021 | Hammond | Prevalence and modes of cannabis use among youth in Canada, England, and the us, 2017 to 2019 |
| 2021 | Harada | A 20-year-old man with e-cigarette or vaping product use-associated lung injury (EVALI) and thrombotic coagulopathy |
| 2021 | Harrell | Longitudinal trajectories of e-cigarette use among adolescents: A 5-year, multiple cohort study of vaping with and without marijuana |
| 2021 | Hotz | Cannabidiol enhances verbal episodic memory in healthy young participants: A randomized clinical trial |
| 2021 | Kahan | Seizure and interictal electroencephalographic (EEG) changes with cannabinoid concentrate use |
| 2021 | Kasson | Using a mixed methods approach to identify public perception of vaping risks and overall health outcomes on Twitter during the 2019 EVALI outbreak |
| 2021 | Kelly | The regulatory and marketing environment surrounding the legalization of retail marijuana and the impact on youth |
| 2021 | Kirkpatrick | #Flavorssavelives: An analysis of twitter posts opposing flavored e-cigarette bans |
| 2021 | Kligerman | Ct findings and patterns of e-cigarette or vaping product use-associated lung injury: A multicenter cohort of 160 cases |
| 2021 | Kostygina | Exploring the discursive function of hashtags: A semantic network analysis of JUUL-related Instagram messages |
| 2021 | Kritikos | Past 30-day marijuana vaping: Prevalence and predictors of use in a nationally representative study of U.S. youth |
| 2021 | Kupelian | E-cigarette or vaping product use-associated lung injury complicated by pulmonary aspergillosis |
| 2021 | Lanza | Tobacco and cannabis poly-substance and poly-product use trajectories across adolescence and young adulthood |
| 2021 | Lee | Predictors of vaping marijuana initiation among us adolescents: Results from the Population Assessment of Tobacco and Health (PATH) study wave 3 (2015-2016) and wave 4 (2016-2018) |
| 2021 | Lee | Histopathological correlation of acute on chronic eosinophilic pneumonitis caused by vaporized cannabis oil inhalation |
| 2021 | Lucero | A 23-year-old man with acute lung injury after using a tetrahydrocannabinol-containing vaping device: A case report |
| 2021 | Masonbrink | Self-reported and documented substance use among adolescents in the pediatric hospital |
| 2021 | McClelland | Case of a 21-year-old man with persistent lung collapse leading to a pericardectomy linked to vape use |
| 2021 | Morean | Demographic and substance use-related differences among high school adolescents who vape cannabis versus use other cannabis modalities |
| 2021 | Moustafa | Adolescent perceptions of e-cigarette use and vaping behavior before and after the EVALI outbreak |
| 2021 | Nguyen | Self-reported changes in cannabis vaping among us adolescents and young adults early in the covid-19 pandemic |
| 2021 | Palamar | Increases in frequent vaping of cannabis among high school seniors in the United States, 2018–2019 |
| 2021 | Palamar | Discordant reporting of vaping of cannabis among high school seniors in the United States |
| 2021 | Parks | Protective factors for nicotine and marijuana vaping among U.S. adolescents |
| 2021 | Pitlick | Evali: A mimicker of covid-19 |
| 2021 | Reboussin | Tobacco and marijuana co-use in a cohort of young adults: Patterns, correlates and reasons for co-use |
| 2021 | Reddy | Characterizing e-cigarette vaping-associated lung injury in the pediatric intensive care unit |
| 2021 | Romm | Mode of marijuana use among young adults: Perceptions, use profiles, and future use |
| 2021 | Salle | Involuntary 5F-ADB-related intoxication following e-cigarette use |
| 2021 | Sarel | Respiratory failure induced by vaping-associated pulmonary injury: Case report of a new entity |
| 2021 | Shah | Torsades de pointes due to excessive marijuana use in a susceptible patient |
| 2021 | Shannon | A mixed-methods study to inform the clarity and accuracy of cannabis-use and cannabis-tobacco co-use survey measures |
| 2021 | Shi | Heterogeneities in administration methods among cannabis users by use purpose and state legalization status: Findings from a nationally representative survey in the United States, 2020 |
| 2021 | Smith | Modes of delivery in concurrent nicotine and cannabis use ("co-use") among youth: Findings from the international tobacco control (ITC) survey |
| 2021 | Trickett | 21-year-old man with fever, night sweats, productive cough, and diarrhea |
| 2021 | Ueno | Age-related differences in cannabis product use |
| 2021 | Wardell | Prevalence and correlates of medicinal cannabis use among adolescents |
| 2021 | Watson | Racial and ethnic differences in marijuana use in e-cigarettes among us youth in 2017, 2018, and 2020 |
| 2021 | Wekon-Kemeni | A gut feeling: Abdominal symptoms as an initial presentation of EVALI |
| 2021 | Wieckowska | Pneumothorax secondary to vaping |
| 2021 | Young-Wolff | Nicotine and cannabis vaping among adolescents in treatment for substance use disorders |
| 2021 | Zuckermann | Changes in cannabis use modes among Canadian youth across recreational cannabis legalization: Data from the compass prospective cohort study |
